# Selection of reference genes for gene expression analysis in *Liriodendron* hybrids’ somatic embryogenesis and germinative tissues

**DOI:** 10.1038/s41598-021-84518-w

**Published:** 2021-03-02

**Authors:** Tingting Li, Weigao Yuan, Shuai Qiu, Jisen Shi

**Affiliations:** 1 Zhejiang Academy of Forestry, Hangzhou, 310023 China; 2 Key Laboratory of Forest Genetics and Biotechnology of Ministry of Education, Co-Innovation Center for Sustainable Forestry in Southern China, Nanjing Forestry University, Nanjing, 210037 China; 3 Research and Development Center, Hangzhou Landscaping Incorporated, Hangzhou, 310020 China

**Keywords:** Developmental biology, Molecular biology, Plant sciences

## Abstract

The differential expression of genes is crucial for plant somatic embryogenesis (SE), and the accurate quantification of gene expression levels relies on choosing appropriate reference genes. To select the most suitable reference genes for SE studies, 10 commonly used reference genes were examined in synchronized somatic embryogenic and subsequent germinative cultures of *Liriodendron* hybrids by using quantitative real-time reverse transcription PCR. Four popular normalization algorithms: geNorm, NormFinder, Bestkeeper and Delta-Ct were used to select and validate the suitable reference genes. The results showed that elongation factor 1-gamma, histone H1 linker protein, glyceraldehyde-3-phosphate dehydrogenase and α-tubulin were suitable for SE tissues, while elongation factor 1-gamma and actin were best for the germinative organ tissues. Our work will benefit future studies of gene expression and functional analyses of SE in *Liriodendron* hybrids. It is also serves as a guide of reference gene selection in early embryonic gene expression analyses for other woody plant species.

## Introduction

Somatic embryogenesis (SE) is a technique that allows the study of early regulatory and morphogenetic events in regenerative systems of higher plants and is also a tool for mass clonal propagation of genetically improved varieties and germplasm maintenance^1–3^. Determining the key regulators of important events during cell differentiation and the major morphogenetic transformational stages, which occur during early embryogenesis, will greatly promote plant phylembryogenesis-related research and tissue culture production. In recent years, with the development of the high-throughput sequencing and microarray technologies, several SE-related microRNAs, lncRNAs and genes have been discovered^4–8^. To elucidate the integrated gene networks of SE processes, the expression levels of these embryo-related genes and their relationships need to be understood. Reverse transcription quantitative real-time PCR (RT-qPCR) has been widely adopted as a standard method for verifying, quantifying and comparing gene expression levels because of its sufficient sensitivity and specificity, as well as being time efficient and technically straightforward compared with other methods. However, not only the biological but also the technological variations, such as the quantity of the initial material, the RNA quality, and the efficiency levels of cDNA synthesis and PCR, may influence the accuracy and reliability of RT-qPCR^9–12^. Consequently, RT-qPCR results need to be normalized by several parallel internal reference genes that participate in the whole experimental workflow along with the genes of interest but have the least amount of variation in their expression levels under various experimental conditions and in different tissues types. The most stable reference genes vary widely in different species, tissues and developmental stages, as well as under different experimental conditions^13–16^. Because there are no universal reference genes for all experiments, it is critical to identify the most stable internal control gene or gene combination prior to normalization in different experiments^10,17^.

*Liriodendron* is an ancient angiosperm genus that belongs to the order Magnoliales and *Liriodendron* hybrids (*Liriodendron chinense* (Hemsl.) Sarg*.* × *L. tulipifera* Linn*.*) is derived from the sexual hybridization between *Liriodendron tulipifera* and *Liriodendron chinense*. Since the SE system of *Liriodendron* hybrids was established in 1993^18^, the origin and development of somatic embryos have been studied and control methods have been improved greatly^19^. Somatic embryos of *Liriodendron* hybrids at different developmental stages can be readily obtained on a large scale from very early stages, which have more or less synchronous patterns. Through the development of sequencing technology, genetic information, such as small RNAs^4^, transcriptomes^20,21^, proteomes^22^ and genomes^23^, for *Liriodendron* have been investigated, and genetic transformation systems have also been established^24^. These characteristics make the SE of *Liriodendron* hybrids a suitable system for investigating the regulation of woody plant SE. Morphological research results and molecular data on SE have been increasingly informative, but limited in gene expression analyses, which are important for studying the molecular regulatory mechanisms. Thus, the establishment of a RT-qPCR detection system is necessary, and suitable internal controls for studying *Liriodendron* SE are required.

**Figure 1:**
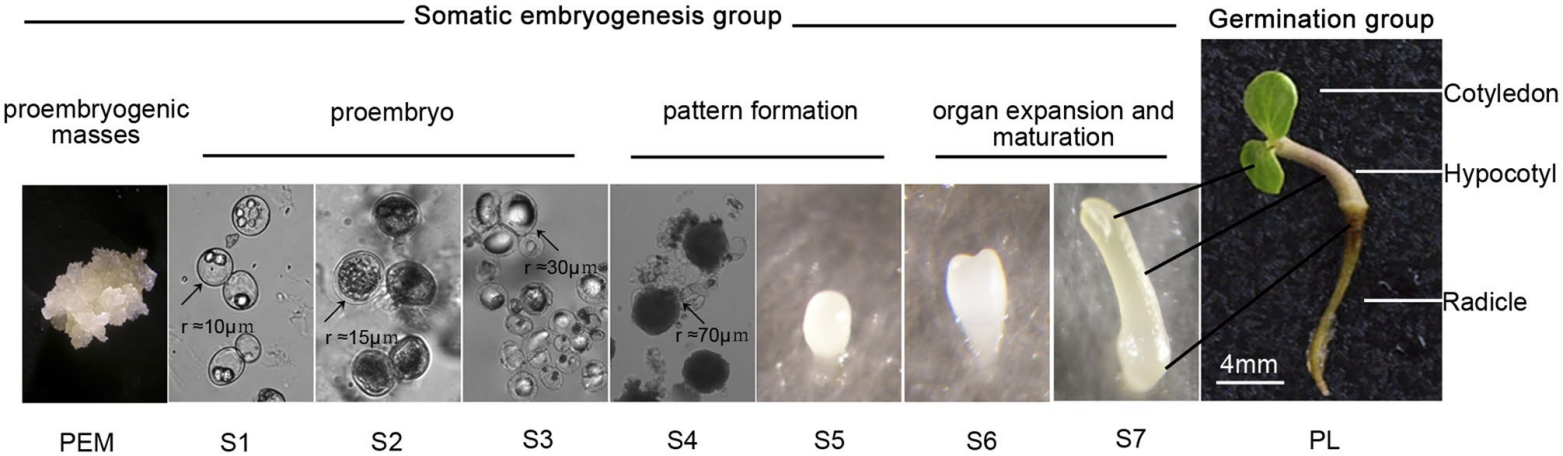
Somatic embryogenesis and germination groups of *Liriodendron* hybrids. The somatic embryogenesis group includes PEM and S1–S7, while the germination group includes cotyledon, hypocotyl and radicle. PEM, proembryogenic masses; S1, proembryogenic single cell stage; S2,embryogenic single cell stage; S3, two to four cell proembryo stage; S4, multicell proembryo stage; S5, globular embryo stage; S6, heart/torpedo -shaped embryo stage; S7, cotyledon embryo stage; PL, plantlet developed from somatic embryo. (The images of PEM and S1 to S7 were provided by the Key Laboratory of Forest Genetics and Biotechnology, Ministry of Education of China, Co-Innovation Center for the Sustainable Forestry in Southern China, Nanjing Forestry University).

Similar to zygotic embryogenesis, the sequence of SE in an angiosperm species can be divided into three phases: proembryo, specific pattern formation and transition to the cotyledonary stage^2,25^. To extend the analysis to the early germination period and different organ tissues of somatic embryo-derived seedlings, we established two experimental groups in this study: somatic embryogenesis group and germination group (Fig. 1). In total, 10 candidate genes for the RT-qPCR normalization analysis expressed in the SE of *Liriodendron* hybrids were compared and evaluated. These included eight widely used reference genes, 18S ribosomal RNA (*18S rRNA*)^26–28^, actin (*ACT*)^27,29^, elongation factor 1-alpha (*EF1a*)^17,28,29^, glyceraldehyde-3-phosphate dehydrogenase (*GAPDH*)^26,29^, histone H1 linker protein (*HIS1*)^17,27,28^, α-tubulin (*TUA*)^17,29^, β-tubulin (*TUB*)^28^ and ubiquitin (*UBQ*)^26–28^, as well as two other potential candidate genes, ribosomal protein L2 encoding gene (*Rpl2*) and elongation factor 1-gamma (*EF1g*).

## Results

### Verification of primer specificity and PCR efficiency analysis

To evaluate the expression stability of candidate reference genes in different SE stages and organs, 11 samples of synchronized *Liriodendron* hybrid embryogenic cultures that were divided into the two test groups were studied. The quality of the total RNA is an important variable factor in RT-qPCR. In our experiment, all the samples were subjected to an on-column DNA removal protocol to eliminate residual genomic DNA. The ratios of A260/A280 and A260/A230 were between 2.01–2.11 and 2.1–2.25, respectively, and the agarose gel electrophoresis also showed good RNA integrity.

The specificity of each primer was verified by dissociation curve analysis, electrophoresis and sequencing. All the primers designed in our experiment showed single peaks in the gradient dilution amplification melt curves (Supplemental data 1). The agarose gel electrophoresis and sequencing results also indicated that the amplicons were of the desired size and were the correct gene sequences (Supplemental data 1). The standard curves analyses revealed slopes between − 3.0 and − 3.5, and the PCR amplification efficiency of each primer pair varied from 93% for *Rpl2* to 109% for *UBQ*. The R^2^ values of the genes varied from 0.95 to 1 (Table 1). These indexes were all in credible intervals, indicating that the test samples had similar PCR efficiencies and that the RT-qPCR data can be used in the following analyses.

**Table 1:** Ranking of the candidate reference genes according to their stability value calculated by NormFinder.

Rank	Somatic embryogenesis group		Germination group	
	Gene name	Stability value	Gene name	Stability value
1	EF1g	0.0024	EF1g	0.0013
2	HIS1	0.0046	ACT	0.0016
3	GAPDH	0.0055	Rpl2	0.0030
4	TUA	0.0063	HIS1	0.0035
5	Rpl2	0.0088	EF1a	0.0062
6	ACT	0.0092	GAPDH	0.0066
7	UBQ	0.0102	UBQ	0.0075
8	EF1a	0.0119	TUB	0.0097
9	TUB	0.0126	TUA	0.0114
10	18S rRNA	0.0210	18S rRNA	0.0190

The input data for NormFinder are supposed to be on a linear scale. The raw Ct values were transformed to linear scale expression quantities using the standard curve or delta-Ct method.

### Expression profiling of reference genes in Liriodendron embryogenesis and germinative tissues

**Figure 2:**
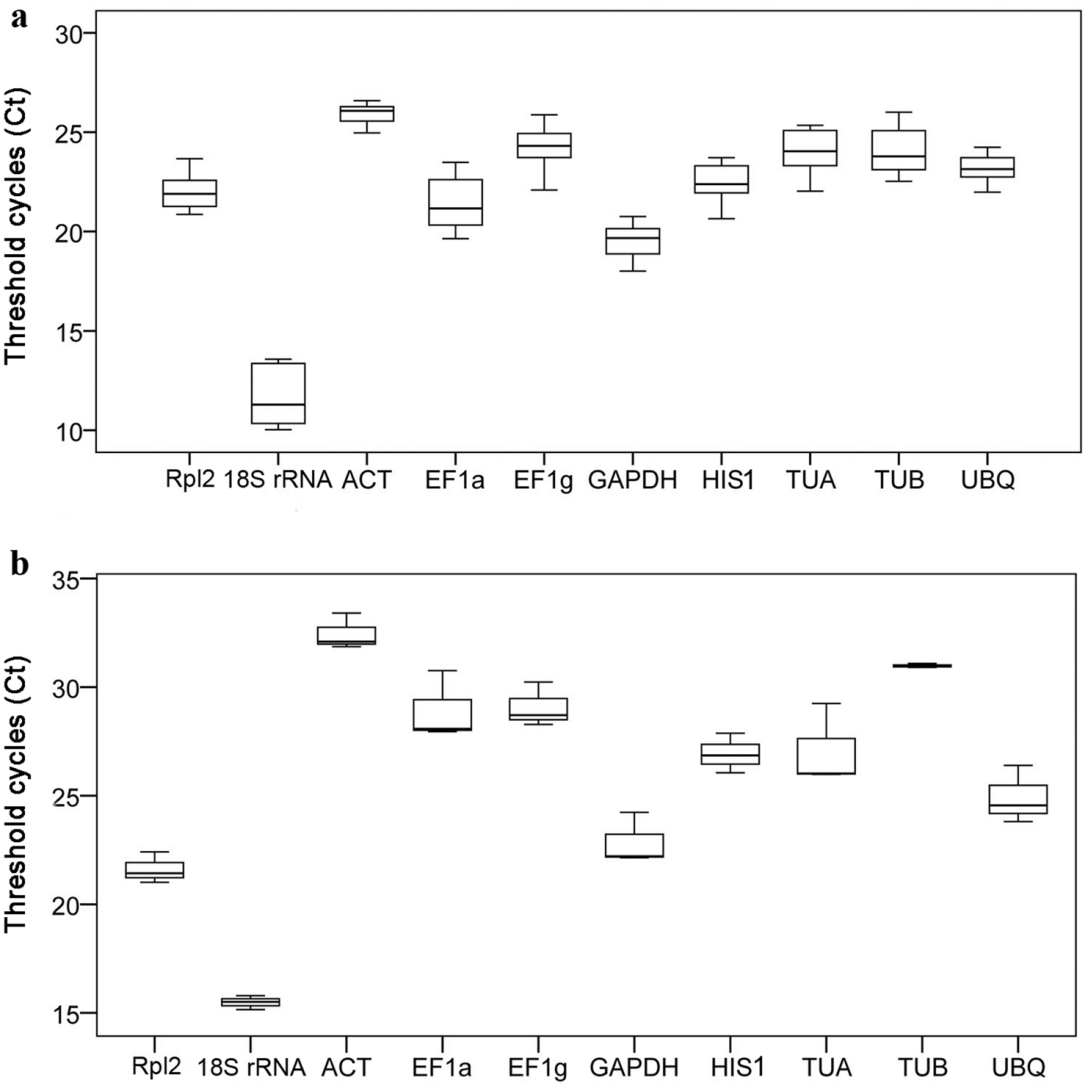
Expression levels of 10 reference genes in *Liriodendron* hybrids as determined by the quantification cycle values, also known as the threshold cycle (Ct) values, in the somatic embryogenesis (**a**) and the germination (**b**) groups. The boxes indicate the 25th and 75th percentiles, the line across each box represents the median, and whisker caps represent the maximum and minimum Ct values.

The expression levels of 10 reference genes in two developmental groups were evaluated by comparing the quantification cycle (Cq) values, also known as the threshold cycle (Ct) values, using box-and-whiskers plots. The gene encoding 18S rRNA had the greatest expression levels, with the lowest Ct values, in the 11 tissues because it is one of the most abundant transcripts, as revealed in a study of embryogenesis in longan trees^28^. The other candidate reference genes’ Ct values in the two test groups were all within moderate boundaries. The median Ct values ranged from 25.92 (*ACT*) to 19.52 (*GAPDH*) and from 32.64 (*ACT*) to 21.88 (*Rpl2*) in the embryogenesis (Fig. 2a) and germination groups (Fig. 2b), respectively.

The range of Ct values in different developmental groups and stages indicated a considerable variability among the 10 candidate reference genes. The least variation in gene expression across the eight embryogenesis samples was found for *ACT* (1.62 cycles), while the most variable was *TUA* (4.16 cycles). In the germination group, the least variable were *ACT* and 18S rRNA (< 2 cycles), while the most variable were *EF1a* and *TUA* (> 4 cycles).

### Gene expression stability analysis and ranking of Liriodendron reference genes

Four different software programs were employed to assess the reference genes stability: geNorm, NormFinder, BestKeeper and ΔCt.

### geNorm analysis

**Figure 3:**
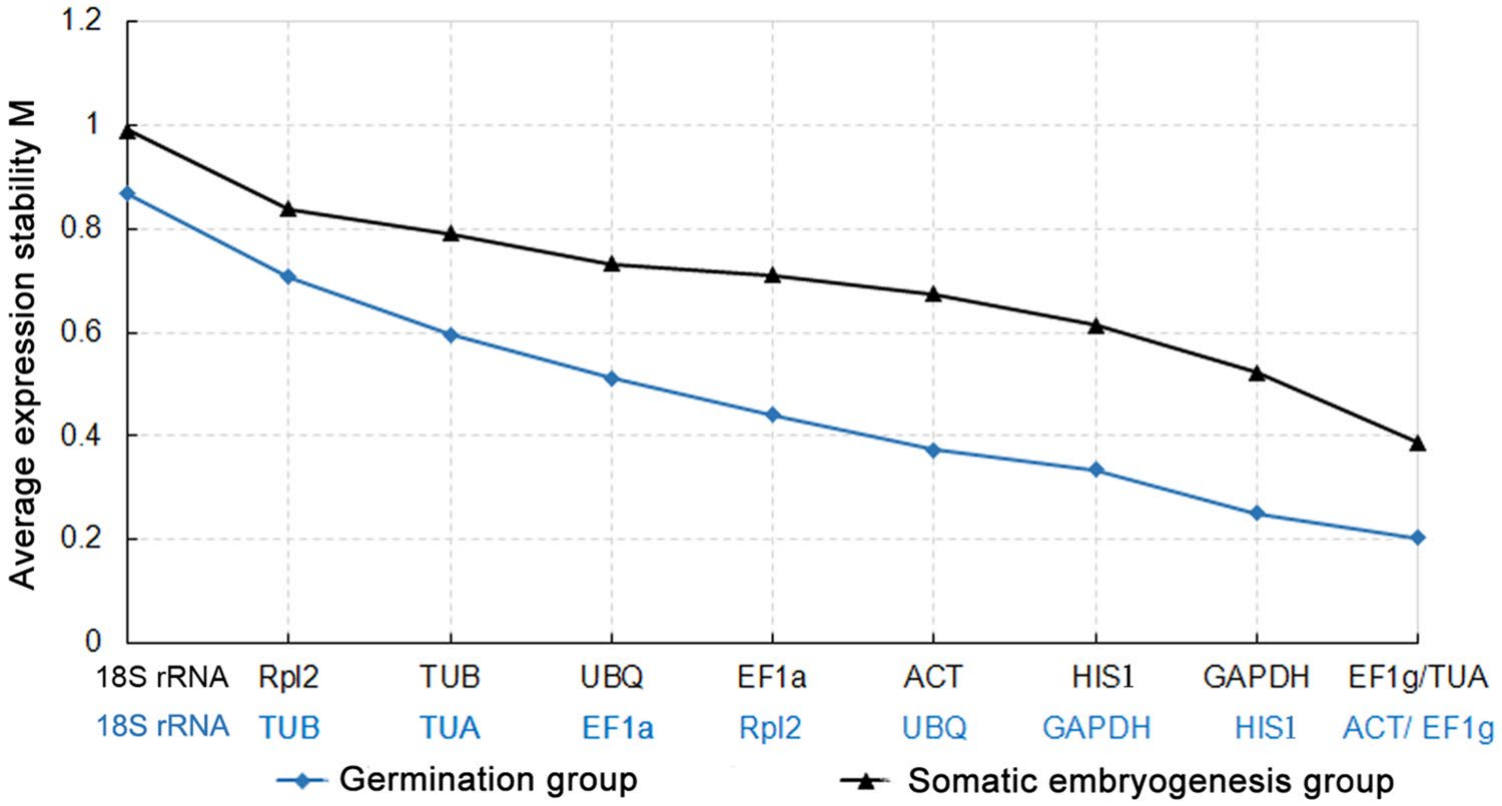
Expression stability and rankings of reference genes in *Liriodendron* hybrids as calculated by geNorm in the somatic embryogenesis (black) and the germination (blue) groups. Genes with the most constitutive expression are indicated on the right side of the graph, with the less stably expressed genes on the left.

**Figure 4:**
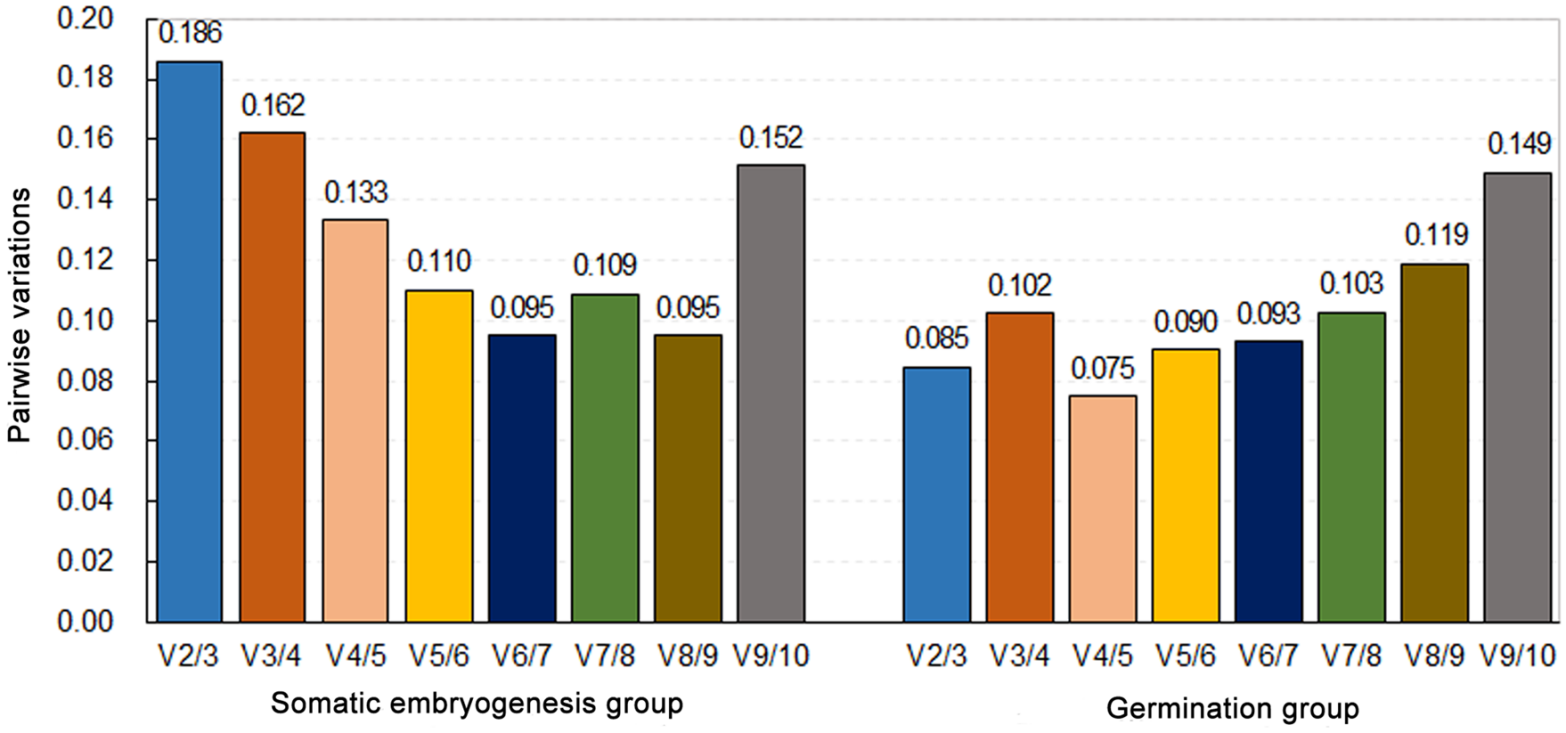
Pairwise variation (V) calculated by geNorm to determine the optimal number of reference genes in the somatic embryogenesis (left) and the germination (right) groups of *Liriodendron* hybrids. A value < 0.15 indicates that the inclusion of an additional reference gene is not required.

geNorm calculates the expression stability value (M) for each candidate gene on the basis of the mean pair-wise variation between all the genes analyzed. Genes with lower M values are more stably expressed. In this study, we used an M value cut-off value of ⩽ 1 to identify the stably expressed genes. The pairwaise variation of two sequential normalization factors (Vn/n + 1 value) determines the number of control genes for normalization, and a threshold value of 0.15 was adopted, as reported by Vandesompele et al^10^. In the somatic embryogenesis subset, the stability of genes (from most to least) was ranked as follows: *EF1g/TUA* > *GAPDH* > *HIS1* > *ACT* > *EF1a* > *UBQ* > *TUB* > *Rpl2* > 18S rRNA (Fig. 3). Because the V4/5 value < 0.15 and V3/4 > 0.15 (Fig. 4), four reference genes with lower M values (*EF1g*, *TUA*, *GAPDH* and *HIS1*) were required for normalization in the embryogenesis group. For the germination subset, which is not the same as the somatic embryogenesis series, the stability of genes (from most to least) was ranked as follows: *EF1g/ACT* > *HIS1* > *GAPDH* > *UBQ* > *Rpl2* > *EF1a* > *TUA* > *TUB* > 18S rRNA (Fig. 3). The V2/3 value was 0.085 (Fig. 4), suggesting that the employment of the two best reference genes, *ACT* and *EF1g*, was enough for normalization. 18S rRNA was found to be the least stable gene in both groups.

### NormFinder analysis

The Normfinder software ranked the set of candidate genes according to the stability of their expression in a given sample set. The lowest stability value represented the most stable gene within the examined gene set. The NormFinder analysis of the two groups is summarized in Table 1. *EF1g* was identified as the most stable gene in both experimental groups. Unlike geNorm, the NormFinder approach takes into account inter- and intra-group variations for normalization factor calculations^30^. When inter-group variations are considered, the combination of the best two genes is provided. Here, they were *EF1g* and *HIS1*. However, the stability value of the two-gene combination was greater than that of the most stable gene, *EF1g* (0.001). In addition, the correlation coefficient (r) when comparing geNorm and NormFinder was not strong (r = 0.644). When only considering intra-group variation in the NormFinder calculations, the results from both algorithms for each experimental set are well correlated. Both NormFinder and geNorm analyses ranked the top stable genes as *EF1g* and *ACT* in the germination series and *EF1g*, *HIS1*, *GAPDH* and *TUA* in the somatic embryogenesis series, although in a slightly different order, with 18S rRNA ranking last in both groups (Table 1).

### BestKeeper analysis

The results of BestKeeper analysis are shown in Table 2. For the embryogenesis group, 18S rRNA and *TUB* were eliminated because of their high standard deviation (SD) values of 1.308 and 1.063, respectively, and coefficient of variance values of 11.154 and 4.406, respectively. *EF1g* was the most stable gene, with the highest correlation coefficient (r) value (0.970, *p* ˂ 0.01). This was followed by *HIS1* (r = 0.905, *p* ˂ 0.01), *TUA* (r = 0.873, *p* ˂ 0.01) and *GAPDH* (r = 0.860, *p* ˂ 0.01). These genes were also the four most stable genes identified by geNorm and NormFinder. For the germination group, *EF1a*, *TUA* and *UBQ* were excluded because they had SD values > 1. *EF1g*, *GAPDH*, *HIS1* and *ACT* correlated well with each other (0.857 < r < 0.958, *p* ˂ 0.01). As in the geNorm and NormFinder analyses, in this group, 18S rRNA and *TUB* ranked last. Because two reference genes were enough for normalization in the germination group, the most reliable gene *EF1g* combined with one of the other three top genes is acceptable.

**Table 2:**
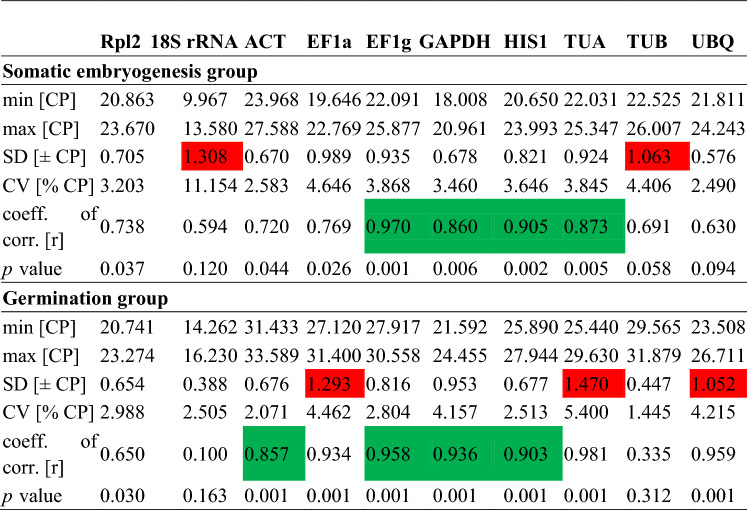
BestKeeper software statistics for reference genes based on Ct values in the somatic embryogenesis and the germination groups.

CP equivalent terminology for Ct, standard deviation (SD; ± CP): the SD of the CP (values greater than 1 are highlighted in red); Coefficient of variance (CV; %CP): the CV expressed as a percentage of the CP level. The correlation between each candidate reference gene and the BestKeeper index was calculated using Pearson’s correlation coefficient (r) (values correlated well are highlighted in green) and the *P* value^31^.

### ΔCt analysis

**Figure 5:**
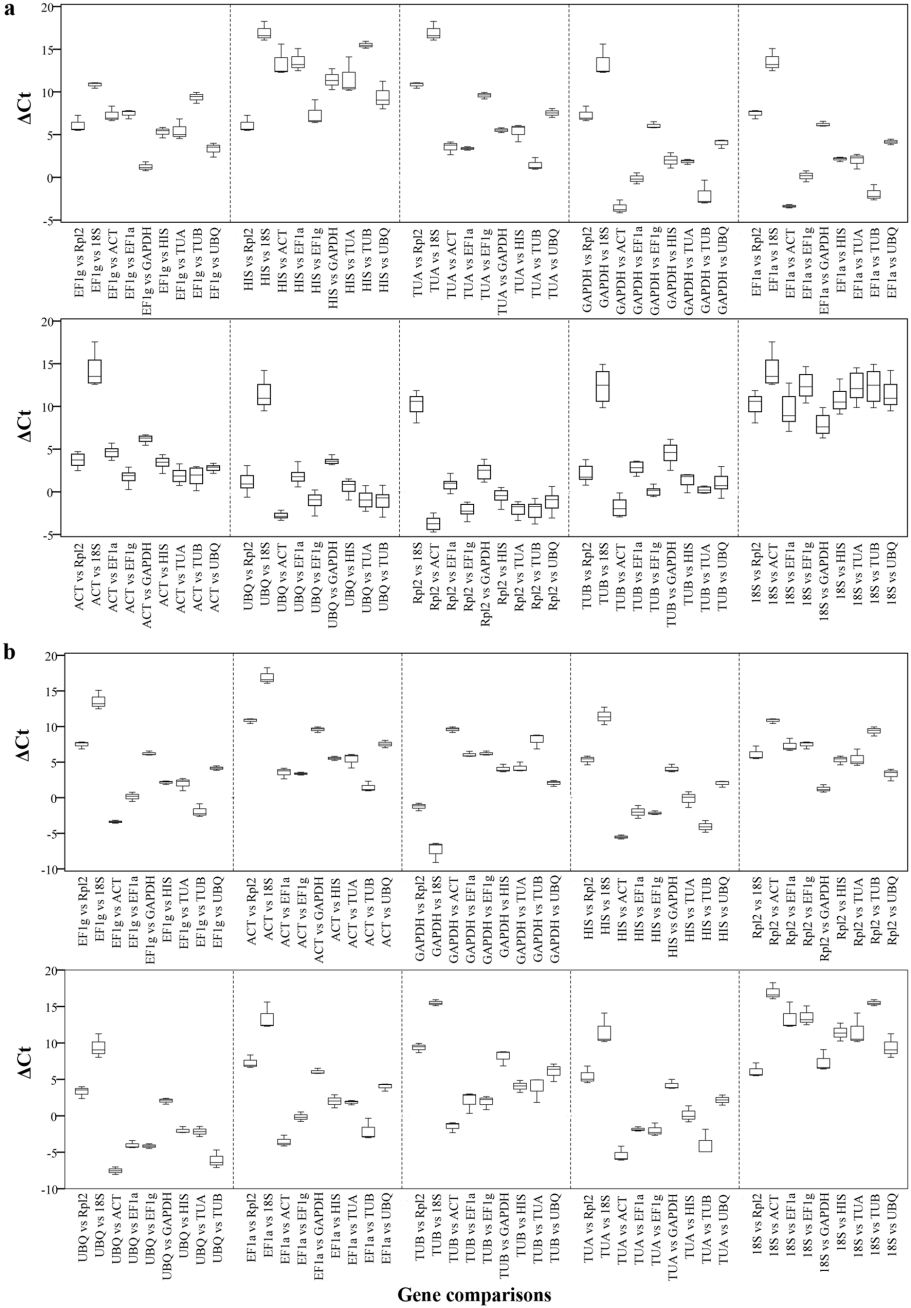
ΔCt method for reference gene selection in *Liriodendron* hybrids. ΔCt variability in reference gene comparisons are shown as medians (lines), 25th percentile to the 75th percentile (boxes) and ranges (whiskers) for all eight samples in the somatic embryogenesis group (**a**) and three samples in the germination group (**b**).

To avoid the influence of the quality of input RNA, we also used the ΔCt approach whereby ‘pairs of genes’ are compared^30,31^. The stability of the reference genes is ranked according to the average SD index. Each gene was compared against the other nine genes, and the appropriate reference genes with low mean variability levels were selected for the two experimental systems. As shown in Fig. 5 and Supplemental data 2, in the somatic embryogenesis group, *EF1g*, *HIS1*, *TUA* and *GAPDH* held the top four positions, with mean SD values of 0.782, 0.856, 0.859 and 0.885, respectively. This result corroborated those of the other three applets. In the germination group, *EF1g* was the most stable genes (mean SD of 0.603), followed by *ACT* (mean SD of 0.607). In both groups, when 18S rRNA was compared with the other nine genes in the respective developmental groups, it showed the greatest amounts of deviation in ΔCt values, indicating that 18S rRNA was the least stable reference gene in ourtest list. In addition, the range of ΔCt values was relatively wider in the somatic embryogenesis group than in the germination group (Fig. 5), indicating that gene expression during somatic embryogenesis stages was more variable than in embryonic germinative organ tissues.

### Reference gene selection

Each method can introduce bias, and it was assumed that a comparison of the different algorithms would allow a more reliable evaluation^32^. Consequently, we used three different Visual Basic applets, geNorm, NormFinder and BestKeeper, and the ΔCt method, to evaluate the stability of 10 reference genes in both the *Liriodendron* SE and germination groups. The gene stability orders of the two experimental groups calculated by four methods are displayed in Table 3. Comparing the outputs obtained from four analytical approaches, as shown in Table 4, the r values indicated that the ranking results of geNorm, NormFinder, and the ΔCt method were significantly correlated at the 0.01 level, and they were inversely associated with the results of BestKeeper at the 0.05 level. In addition, their correlations in the germination group were more relevant than those in the somatic embryogenesis group. The correlation analyses indicated that the results of the four approaches tended to coincidence, although each approach was based on a different algorithm.

**Table 3:** Expression stability of reference genes analyzed by geNorm, NormFinder, BestKeeper and delta Ct.

Stability rank	Somatic embryogenesis group				Germination group			
	geNorm	NormFinder	BestKeeper	ΔCT	geNorm	NormFinder	BestKeeper	ΔCT
1	EF1g	EF1g	EF1g	EF1g	EF1g	EF1g	EF1g	EF1g
2	TUA	HIS1	HIS1	HIS1	ACT	ACT	GAPDH	ACT
3	GAPDH	GAPDH	TUA	TUA	HIS1	HIS1	HIS1	GAPDH
4	HIS1	TUA	GAPDH	GAPDH	GAPDH	Rpl2	ACT	HIS1
5	ACT	Rpl2	EF1a	EF1a	UBQ	EF1a	EF1a	Rpl2
6	EF1a	ACT	Rpl2	ACT	Rpl2	GAPDH	UBQ	UBQ
7	UBQ	UBQ	ACT	UBQ	EF1a	UBQ	TUA	EF1a
8	TUB	EF1a	TUB	Rpl2	TUA	TUB	Rpl2	TUB
9	Rpl2	TUB	UBQ	TUB	TUB	TUA	TUB	TUA
10	18S rRNA	18S rRNA	18S rRNA	18S rRNA	18S rRNA	18S rRNA	18S rRNA	18S rRNA

**Table 4:** Correlations of the four algorithms’ results (r values).

Algorithm	Somatic embryogenesis group				Germination group			
	geNorm	NormFinder	BestKeeper	ΔCT	geNorm	NormFinder	BestKeeper	ΔCT
geNorm	–	0.876**	− 0.870**	0.814**	–	0.910**	− 0.776	0.957**
NormFinder	0.876**	–	− 0.870**	0.916**	0.91**	–	− 0.689*	0.939**
BestKeeper	− 0.870**	− 0.870**	–	− 0.746*	− 0.776	− 0.689*	–	− 0.711*
ΔCT	0.814**	0.916**	− 0.746*	–	0.957**	0.939**	− 0.711*	–

*, **Significantly different at the 0.05 and 0.01 levels, respectively.

The pairwise comparison approach (geNorm) not only produces a gene ranking but provides a rational for determining the minimum number of genes required for accurate normalization. According to the Vn/n + 1 value, four reference genes in the somatic embryogenesis subset and two in the germination subset were needed. Based on the results of all four statistical methods, the most stable combination of reference genes for SE stages was *EF1g*, *HIS1*, *GAPDH* and *TUA*, while for the germinative organ tissues the most stable reference gene was *EF1g* and the optimal reference combination was *EF1g* and *ACT*.

## Discussion

Normalization is crucial for obtaining reliable gene expression data by RT-qPCR. RT-qPCR studies in SE are difficult because, from a single somatic cell to a mature embryo, plant somatic embryos go through cell differentiation, organ formation and maturation, which are accompanied by complex networks containing many dynamic developmental, biochemical and metabolic processes. A large number of genes are variably expressed in space and time, and to different extents. They are also affected by changes in extracellular and external environmental factors^1,33^. To date, there have been several reports on selecting suitable internal controls for plant SE, such as in maritime pine^26^, cotton^27^, longan tree^28^, citrus^34^ and conifer species^17^, but these studies either did not identify stable controls, or the study materials did not cover the complete SE process, lacking especially in the early somatic embryogenesis stages. Additionally, some of them studied gymnosperms which have SE processes that are not quite the same as those of angiosperms^2^. In this study, 10 candidate genes and four statistical applets (geNorm, NormFinder, BestKeeper and the ΔCt method) were selected to perform expression stability analyses of two test groups at eight developmental stages during SE and in three different tissues in the following germination phase in *Liriodendron* hybrids. The results suggest that the combination of *EF1g*, *HIS1*, *GAPDH* and *TUA* is optimal for the normalization of gene expression in the embryo developmental series, while *EF1g* or *EF1g/ACT* is a stable reference gene(s) in the germinative tissues.

Elongation factor expression is correlated with the synthesis of proteins during cell cycle and elongation in some cells. *EF1a* was recommended as a stable reference gene in embryogenic cell suspensions of *Coffea arabica*^35^ and in olive mesocarp tissues^36^. In our research, *EF1a* ranked in the middle position in both SE and plantlet groups, while *EF1g* was the most stable reference genes in both groups. Twardowski and Legocki^37^ and Dell'Aquila et al.^38^ demonstrated that some elongation factors play important roles in plant cells at early stages of seed germination, and the properties of EF1 are correlated with ageing-related phenomena. We speculated that *EF1g* and *EF1a* are expressed differently in different stages and tissues, and *EF1a* seemed more variable because of some molecular and functional properties.

Histones are the chief protein components of chromatin. They package and order the DNA into nucleosomes. The Histone H1 family is one of the five main Histone families, it is half the size of the other four histones and can be expressed in different or overlapping tissues and developmental stages. *HIS1* variants are partially redundant and vary little among different experiments^39^. Glyceraldehyde -3-phosphate dehydrogenase, as an abundant glycolytic enzyme, participates in the cell metabolism and several non-metabolic processes, including transcriptional activation, cell apoptosis^40^, endoplasmic reticulum to Golgi vesicle shuttling, and fast axonal or axoplasmic transport^41^. These cellular energy-associated actions occur consistently during all the developmental stages of *Liriodendron* SE. Although, in some cases, *GAPDH* is an inappropriate endogenous control^26,35^ and it was not the most stable gene in our test, owing to the complex SE-related mechanisms, it is a necessary member of the control combination.

Actin and tubulin are members of cytoskeleton and are ubiquitous in plant cells. Actin fulfils a variety of specific roles, such as in cell division and elongation, cell wall development, cell movement and developmentally regulated programmed cell death^42^. It has been reported as a housekeeping gene and used as a control in RT-qPCR analyses, but not in the analyses of any tissues. In the non-fiber tissues of cotton, it was the most variable reference gene^27^. In our study, the stability of *ACT* in the SE group ranked in the middle position, but it was relatively stable in the germination group. The *ACT* gene family is large, and different members play different roles in distinct tissues and developmental stage. Schwarzerová et al.^42^ reported that some actin isoforms could even control the speed and synchrony of development in the SE of spruce. The tubulin genes are similar. Although all the tubulins in the same organism appear able to participate in all the major functions, some tubulins are tissue and stage-specific. α-Tubulin and β-tubulin groups exhibit major differences in their net electric charges, dipole moments and dipole vector orientations, and these differences may influence their functional characteristics^43^. In our study, in the SE group, the expression of *TUA* was relatively stable but *TUB* was variable, while in the germination group, both of them ranked among the lower positions.

During SE in *Liriodendron* hybrids, 18S rRNA was classified as the least stable gene analyzed by all the applets and, therefore, is not suitable as an internal control. This result is in accordance with other reference gene selection reports related to SE^26–28^. We speculated that 18S rRNA would not be selected as an internal control gene in this test because of its high content in total RNA, which led to a small Ct value (˂ 15 cycles) in the RT-qPCR.

By accumulating data from transcriptome, genome and microRNA sequencing or Chip technology, superior reference genes might be found, like snoR14 and snoRD25 in the SE of citrus^34^, *SAND*, *TBP* and other expressed genes in brinjal fruit developmental stages^44^, dlo-miR24 in the SE of longan^45^, miR167-1_2, miR11-1, miR159-1 and miR168-1 in the seed development of *Brassica napus*^46^. Our research group has also carried out microRNA chip and sequencing experiments, but the data did not reveal any microRNAs that were suitable to use as controls in the early stages of *Liriodendron* hybrid SE^4^. Further testing of more reference genes should be performed if a more efficient internal control is required, but, at present, the use of more than one reference gene as controls could result in the most reliable gene transcription analyses.

In summary, among the 10 candidate reference genes studied, we recommend the combination of *EF1g/HIS1/GAPDH/TUA* genes for normalization of qPCR analyses in the somatic embryogenesis group and *EF1g* or *EF1g/ACT* for the germination group. The commonly used 18S rRNA should be avoided. The results provide guidelines for the selection of reference genes for the normalization of qPCR in future *Liriodendron* hybrid transcriptomic and microRNA studies involving somatic embryogenesis and germination-related tissues, and these guidelines may also be useful in SE gene expression studies of other woody plants.

## Materials and methods

### Plant materials and treatments

In this study, we established two separate experimental groups: somatic embryogenesis and germination. The somatic embryogenesis group started with proembryogenic masses (PEMs) and finished at the cotyledonary stage (S7). It covered the whole embryonic phase prior to germination. In this group, the plant materials were cultured under dark conditions, and each stage had an obvious morphological transformation (Fig. 1). The mature somatic embryos (S7) were then transferred to 16-h (light)/8-h (dark) conditions, and the subsequently developed cotyledon, hypocotyl and radicle were used as the germination group.

Synchronized *Liriodendron* embryogenic cultures at different developmental stages (Fig. 1) were obtained following previously published methods^4,47^. The cotyledon, hypocotyl and radicle were harvested from the developed somatic plantlets when the cotyledon just opened (Fig. 1). All stages of somatic embryos and organ tissues were placed in cryostorage vials immersed in liquid nitrogen and then stored at – 80 °C until used.

### Total RNA extraction and cDNA synthesis

Total RNA was isolated and purified from each SE stage using a Total RNA Purification kit (Norgen Biotek Corporation, Canada), according to the manufacturer’s instructions with the on-column DNA removal protocol^4^. The purity and integrity of the extracted RNA were checked using a NanoDrop 2000/2000C spectrophotometer (Thermo Scientific, USA) and 1.5% agarose gel electrophoresis with ethidium bromide staining. First-strand cDNA was synthesized by reverse transcribing 1.5 μg of total RNA with random primers and an oligo dT primer in a final reaction volume of 20 µl using the SuperScript III First-Strand Synthesis System for RT-PCR kit (Invitrogen, USA) according to the manufacturer’s instructions.

### Primer design and verification of amplified products

We selected 10 reference genes that are commonly used as controls for plant gene expression studies and had relatively stable expression levels during plant embryogenesis^26–28^. To identify *Liriodendron* homologs of these genes, we first searched the *Liriodendron* protein/nucleotide database in NCBI (*Rpl2*, 18S rRNA, *ACT* and *EF1a*) and ChromDB (*HIS1*). The other genes were identified in *Liriodendron* EST databases using TBLASTN with corresponding Arabidopsis protein sequences as the query. Selected *Liriodendron* ESTs were then used as query for BLASTX searches of Arabidopsis (*EF1g*, *GAPDH*, *UBQ* and *TUB*) and Populus (*TUA*) protein databases. The primers were designed with melting temperatures of 58–62 °C using Primer Premier5.0 software (PREMIER Biosoft International, USA) and Oligo 6 (Molecular Biology Insights, USA) according to the obtained mRNA sequences. All the primers were synthesized by Invitrogen.

PCR products obtained using the designed primers were first verified by electrophoresis on 2% agarose gels to have single bands of the expected sizes (Supplemental data 1). To confirm the amplicon sequences, PCR was performed on the cDNAs. Follow the instructions of Ex Taq DNA polymerase (TaKaRa, Japan), reactions were performed with 400 nM of each primer, 1 U Ex Taq DNA polymerase, 200 μM dNTPs (TaKaRa, Japan) and 10 ng of cDNA in a total volume of 20 μl. Amplifications were performed with the universal cycling conditions(95 °C for 5 min, followed by 35 cycles at 94 °C for 30 s, 55 °C for 30 s and 72 °C for 1 min)^36^. The amplified products were purified using an AxyPrep PCR Cleanup Kit (Axygen Biosciences, USA) according to the manufacturer’s instructions and subsequently cloned into the pMD19-T vector (TaKaRa). Positive colonies for each cDNA were sequenced by Invitrogen. The sequence files were analyzed using DNAMAN and verified by the nucleotide BLAST program at NCBI (http://blast.ncbi.nlm.nih.gov/). All the reference genes’ putative functions, accession numbers, primer sequences and amplicon sizes are provided in Table 5.

**Table 5:** Descriptions of *Liriodendron* hybrids’ candidate reference genes and associated qPCR analyses.

Symbol	Gene description/function	GenBank no	Primer sequences	Tm (°C)	Amplicon (bp)	Slop	R^2^	E (%)
Rpl2	Ribosomal protein L2/Protein synthesis	AF123796	F:5′-ACACCAATCCATCCCGAACT-3′	79.73	93	− 3.494	0.999	93.3
			R:5′-TCCTGGCGTCGAGCTATTT-3′					
18S rRNA	18S ribosomal RNA/Constituent of the small ribosomal subunit	FR870008	F:5′-ATTTCTGCCCTATCAACTTTCG-3′	82.42	183	− 3.466	0.998	94.5
			R:5′-TTGTTATTTATTGTCACTACCTCC-3′					
ACT	Actin/Cytoskeletal structural protein	GQ246181	F:5′-CTCGGCTGTGGTTGTGAAG-3′	82.32	160	− 3.469	0.996	94.2
			R:5′-TGGTGTGATGGTTGGTATGGG-3′					
EF1a	Elongation factor 1-alpha /Protein synthesis	FR870012	F:5′-ATCATGAATCACCCAGGACA-3′	80.27	147	− 3.017	0.954	104.5
			R:5′-TTCAAGAACTTGGGCTCCTT-3′					
EF1g	Elongation factor 1-gamma/Protein synthesis	FD496784	F:5′-ATCCGGGATTCACTTGGATA-3′	75.85	114	− 3.349	0.998	98.9
			R:5′-CGTCACCCATAACTTTGCAC-3′					
GAPDH	Glyceraldehyde-3-phosphate dehydrogenase/metabolic function	FD495585	F:5′-ACAACTAACTGCCTTGCTCCTT-3′	83.5	274	− 3.263	0.997	102.5
			R:5′-AGTCAGATCCACCACCGAAA-3′					
HIS1	Histone H1 linker protein/Compacting DNA strands and Chromatin regulation	CO995299	F:5′-AAGCCGAAGAAGGCGAAAGC-3′	81.93	100	− 3.137	0.986	108.3
			R:5′-TCTCGAAGTAGGGCGGATGG-3′					
TUA	a-Tubulin/Cytoskeletal structural protein	FD499140	F:5′-TGACTGGAGCATAAGATGAAAGC-3′	80.46	153	− 3.300	0.983	100.9
			R:5′-CCAATCTCAACCGCCTCG-3′					
TUB	β-Tubulin/Cytoskeletal structural protein	FD493593	F:5′-TCTCCGCCTCCGTAAACTC-3′	80.64	140	− 3.330	0.991	99.7
			R:5′-ATTCATTGGGAACTCAACATCG-3′					
UBQ	Ubiquitin/Protein degradation	CK755254	F:5′-GGCATTCCACCAGACCAGC-3′	82.49	131	− 3.118	0.998	109.3
			R:5′-TGCATCCCGCCCCTCAAT-3′					

F forward primer, R reverse primer, Tm melting temperature, Slop standard curve slope, R^2^ regression coefficient, E amplification efficiency.

### RT-qPCR and statistical analyses

The RT-qPCRs were performed on an ABI 7500 Real-Time PCR System with software (PE Applied Biosystems, USA). Each amplification was performed in a 20 µl final volume that contained 10 μl of Power SYBR Green Master Mix (Applied Biosystems), 0.8 μl of each specific primer pair at 100 nM; 1.0 μl of 5 × diluted cDNA template and 7.4 μl of ddH_2_O. All the PCRs were performed under the following conditions^48^: 2 min at 50 °C, 10 min at 95 °C, and 40 cycles of 15 s at 95 °C and 1 min at 60 °C in 96-well optical reaction plates (Applied Biosystems, USA). The specificity of each reaction was verified by a melting curve analysis (65 °C to 95 °C) after 40 cycles and 2% agarose gel electrophoresis (Supplemental data 1). Three biological replicates for each sample were used for RT-qPCR analysis. Three technical replicates and no template controls for every primer pair were performed for each biological replicate.Four-point standard curves of a fivefold dilution series (1:1 to 1:125) from pooled cDNA were used to calculate PCR efficiency levels^28^ (E) using the following equation: E = (10^−1^/slope^−1^) × 100. The calculated slopes of the standard curve, coefficients of determination, R^2^, and PCR efficiencies are shown in Table 5.

The data obtained were converted into correct input files, according to the requirements of the software, and analyzed using three different Visual Basic applets: geNorm version 3.4^10^, NormFinder (version 0.953)^30^ and BestKeeper (version 1.0)^31^. The comparative ΔCt approach^49^ was also performed using IBM SPSS Statistics Version 19 software (IBM, USA).The geNorm VBA applet can calculate an M-value describing the average pairwise variation of each reference gene in comparison with all the other candidates and ranks the genes according to their expression stability. Lower M values reflect the greater stability of the reference genes. This applet also evaluates the pairwise variation (V_n_/V_n+1_) to determine the optimal number of genes. If the variation is low (Vn/Vn + 1 < 0.15), then this suggests that the added reference gene is not required for the calculation of the normalization factor and thus can be excluded^10,46,50^. The InputData of geNorm were the normalized relative quantities transformed from Ct values using the ΔCt method. As in geNorm, the InputData of NormFinder were log transformed into a linear scale. The NormFinder uses an ANOVA-based model to calculate a stability value, and candidate genes with minimal intra- and inter-group variations have the lowest stability values and are, therefore, ranked at the top^30,46,51^. The BestKeeper algorithm determines the most stably expressed genes based on three variables: SD, coefficient of variance and r value. Genes with SDs greater than 1 are considered unacceptable. BestKeeper also ranks gene stability by estimating the r value, the closer it is to 1, the more stable the gene expression^31,52^. The ΔCt method calculates the relative expression of ‘pairs of genes’ within each sample to identify useful reference genes. The stability of the reference gene is ranked according to a ‘process of elimination’ technique, in which genes are compared to one another, and the less variability in the ΔCt among different samples, the more stably the reference gene is expressed^49,51^.

## Supplementary Information

Supplementary Data 1.

Supplementary Data 2.

## Abbreviations

SE: Somatic embryogenesis
Rpl2: Ribosomal protein L2
18S rRNA: 18S ribosomal RNA
ACT: Actin
EF1a: Elongation factor 1-alpha
EF1g: Elongation factor 1-gamma
GAPDH: Glyceraldehyde-3-phosphate dehydrogenase
HIS1: Histone H1 linker protein
TUA: α-Tubulin
TUB: β-Tubulin
UBQ: Ubiquitin
RT-qPCR: Quantitative real-time reverse transcription PCR
