# Supplementary material for: Selection of reference genes for gene expression analysis in Liriodendron hybrids’ somatic embryogenesis and germinative tissues
Source: Sci Rep. 2021 Mar 2;11:4957. doi: 10.1038/s41598-021-84518-w (PMC7925589; doi:10.1038/s41598-021-84518-w)

Selection of reference genes for gene expression analysis in Liriodendron hybrids’ somatic embryogenesis and germinative tissues

Tingting Li[1]*,Weigao Yuan[1],Shuai Qiu[2,3],Jisen Shi[2]

[1]Zhejiang Academy of Forestry, Zhejiang 310019, China

[2]Key Laboratory of Forest Genetics and Biotechnology, Ministry of Education of China, Co-Innovation Center for the Sustainable Forestry in Southern China, Nanjing Forestry University, Nanjing 210037, China

[3]Hangzhou Landscaping Incorporated, Hangzhou 310020, China

*Corresponding author: Tingting Li: tingtingli71@163.com

Supplemental data 1. Agarose gel electrophoresis and melting curve of reference genes.

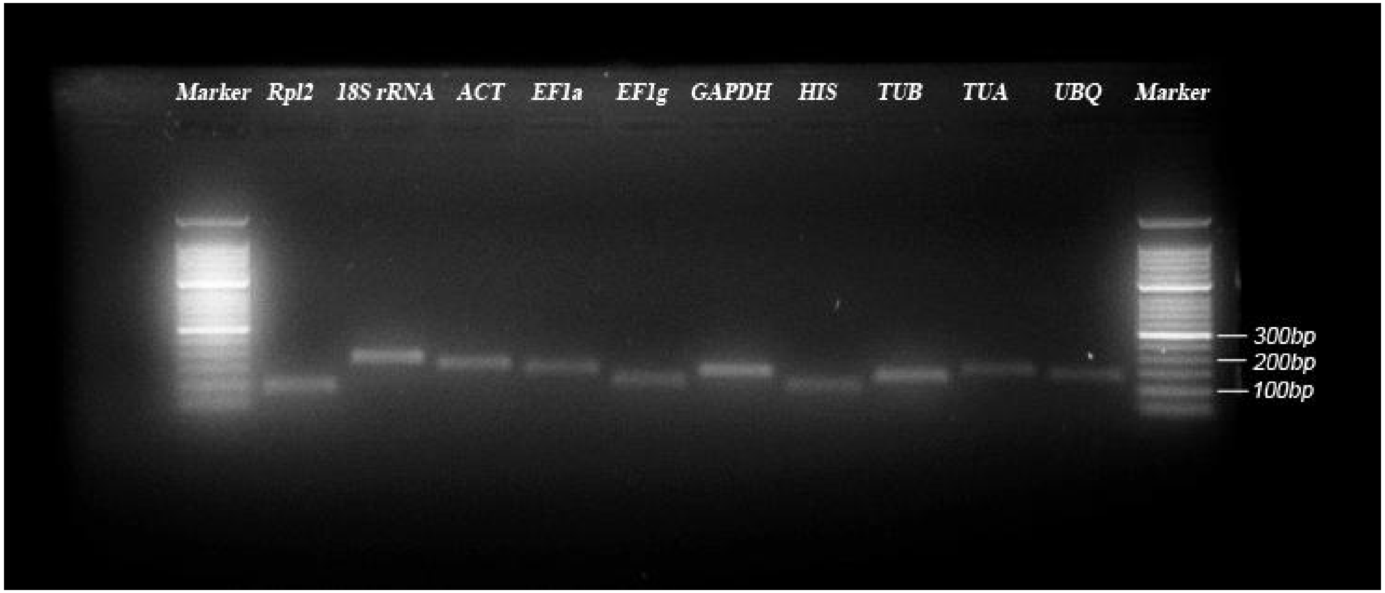

Gradient dilute mixed samples | Total development stages

Rpl2

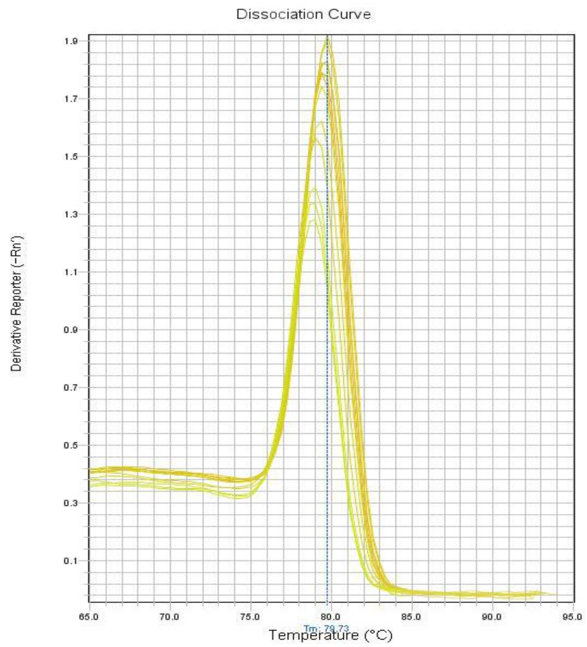

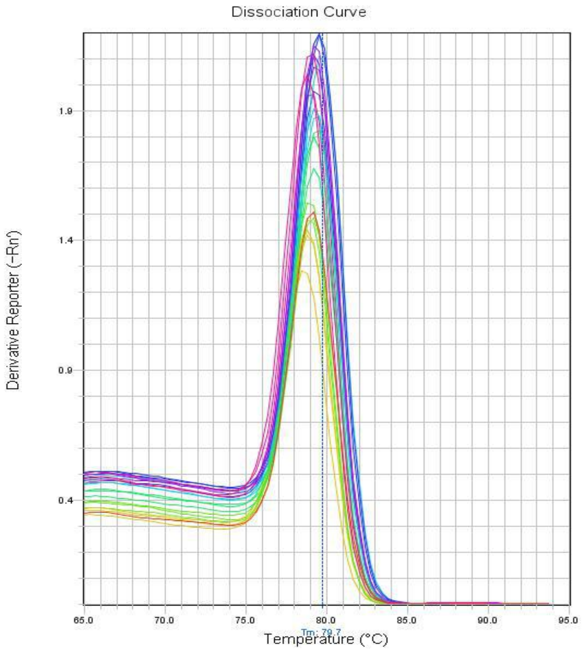

18S rRNA

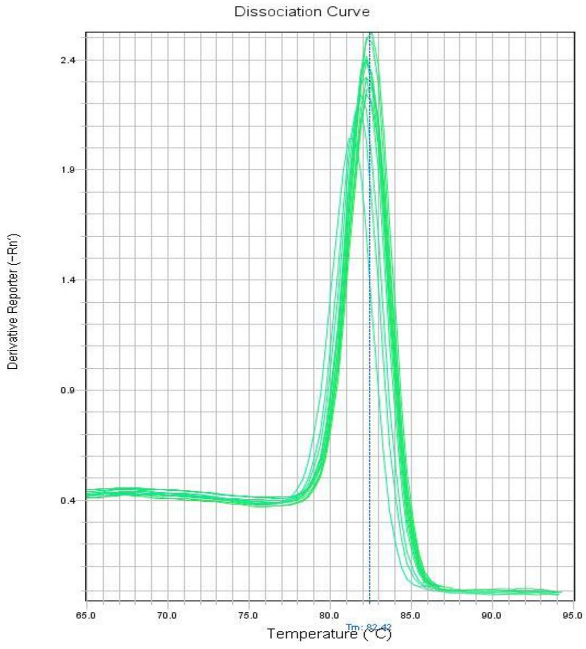

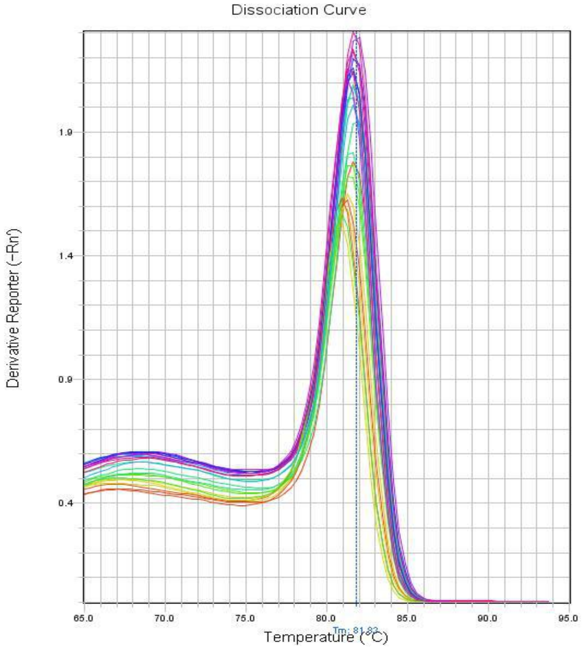

Gradient dilute mixed samples | Total development stages

ACT

EF1a

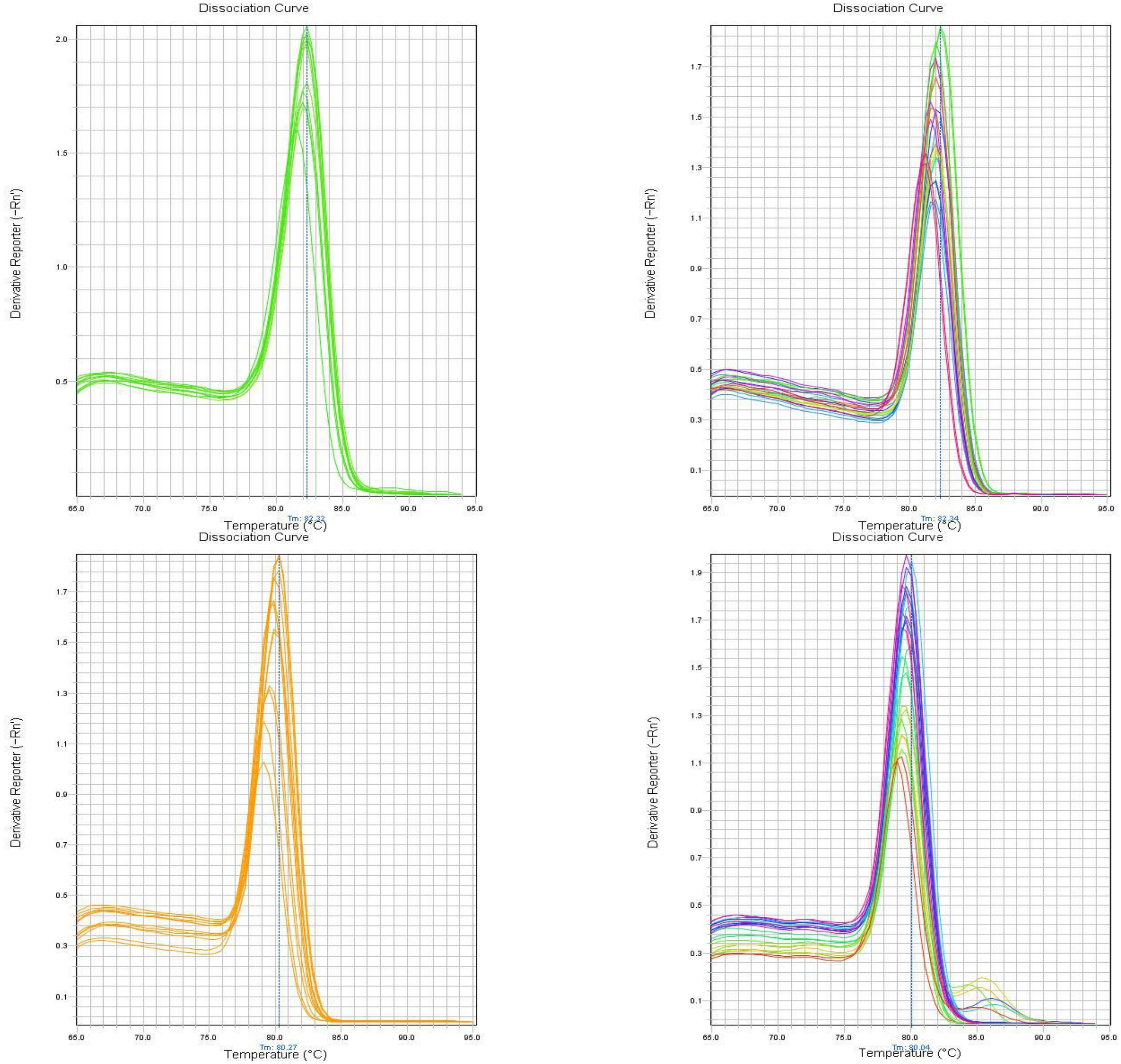

Gradient dilute mixed samples

Total development stages

EF1g

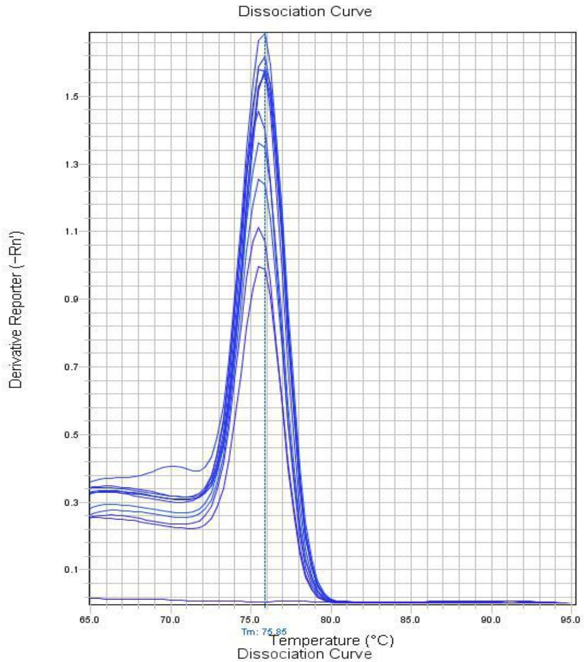

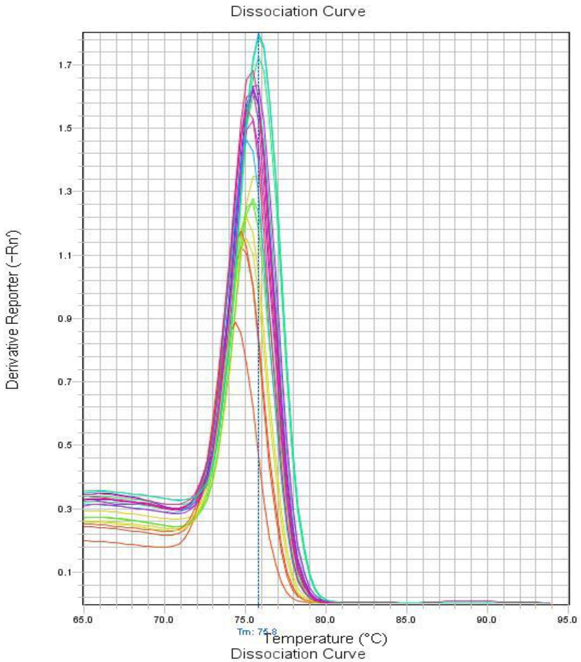

GAPDH

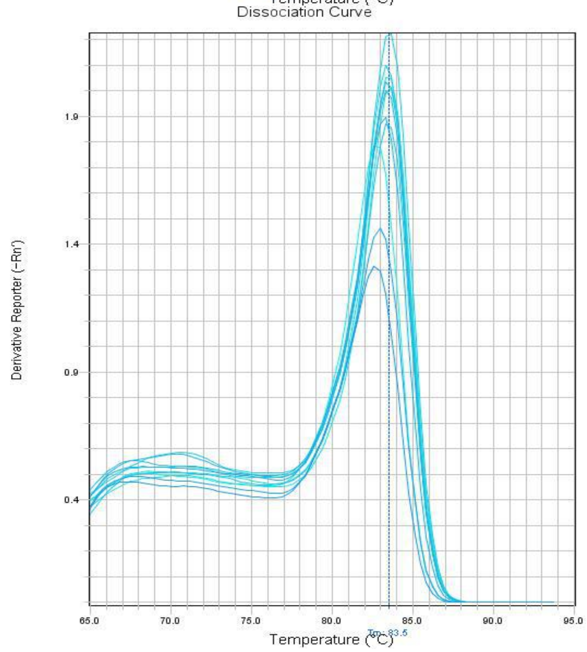

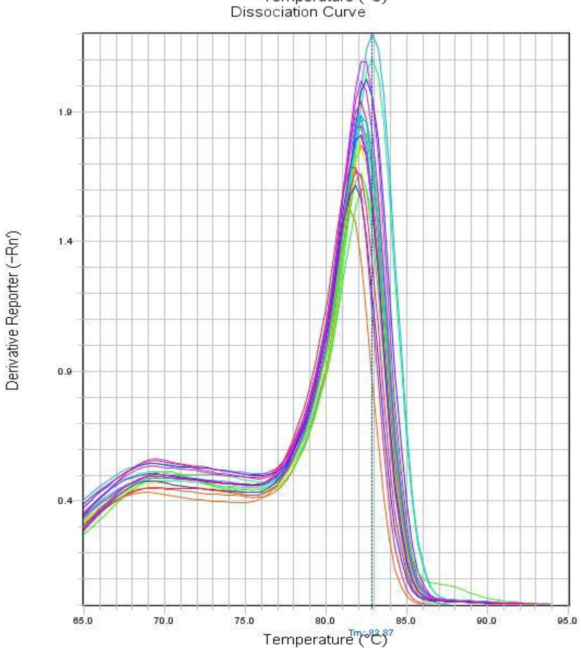

Gradient dilute mixed samples | Total development stages

HIS

TUA

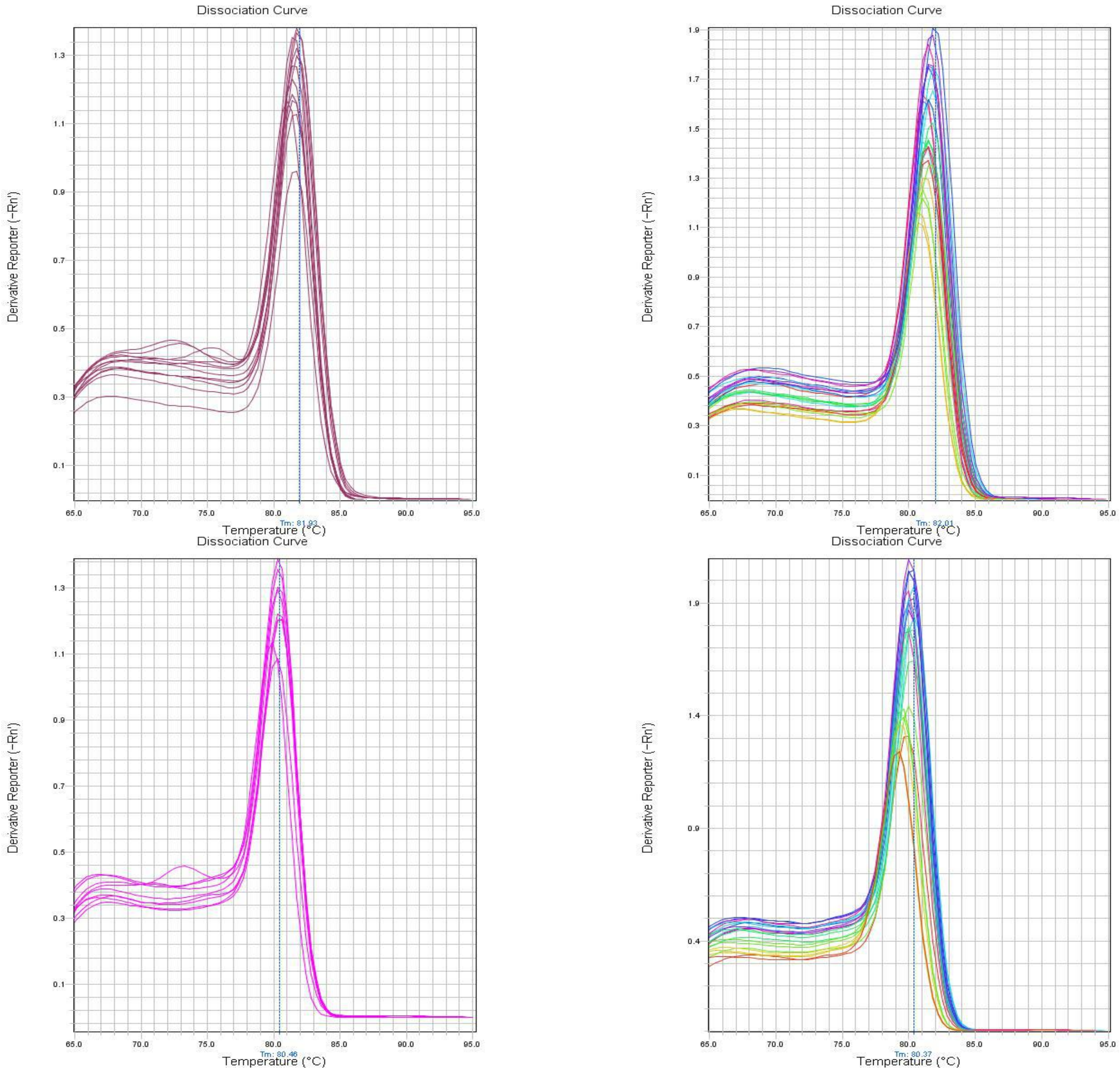

Gradient dilute mixed samples

Total development stages

TUB

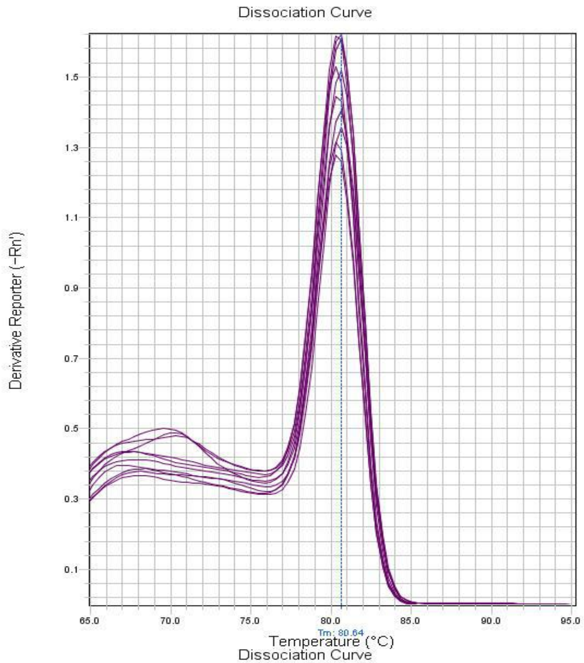

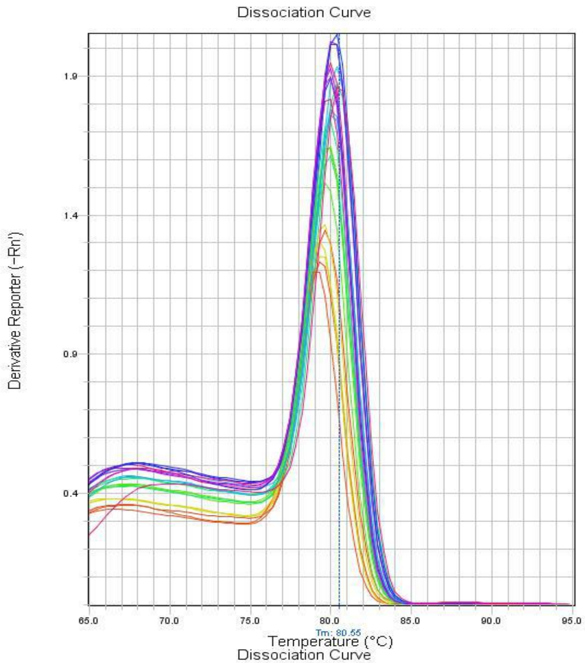

UBQ

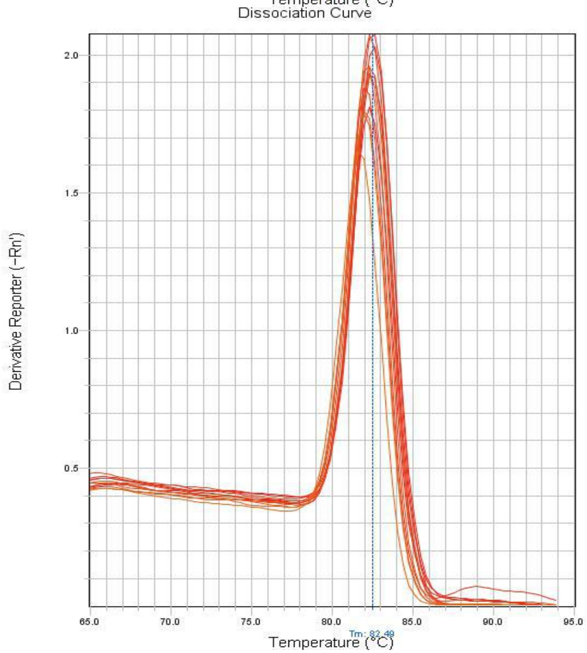

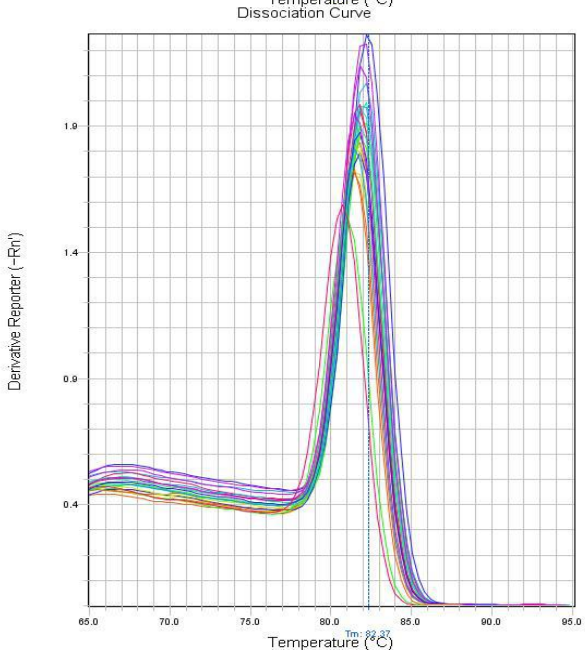

Supplement: Supplementary file 1 — Supplementary Data 1. [file 41598_2021_84518_MOESM1_ESM.pdf]
